# Strengthening the Public Health Partnership and Telehealth Infrastructure to Reduce Health Care Disparities

**DOI:** 10.1089/pop.2022.0166

**Published:** 2022-12-16

**Authors:** Jie Chen, Merianne Rose T. Spencer, Portia Buchongo

**Affiliations:** ^1^ Department of Health Policy and Management, School of Public Health, University of Maryland, College Park, Maryland, USA.; ^2^ The Hospital and Public Health InterdisciPlinarY Research (HAPPY) Lab, School of Public Health, University of Maryland, College Park, Maryland, USA.

**Keywords:** public health system, telehealth postdischarge, racial and ethnic disparities, aging health

## Abstract

The COVID-19 pandemic has underscored the urgency to focus on the essential value of public health systems (PHSs) in fostering health equity across the US health care delivery system. PHS integration and care coordination can be successfully achieved through health information technology systems. The objective of the study was to examine the association between PHS partnerships (PHSPs), telehealth postdischarge, and racial and ethnic disparities in health care. The analysis used 2017 Centers for Medicare and Medicaid Services Medicare 100% inpatient claims data, the Medicare Beneficiary Summary File, the American Hospital Association Annual Survey, and the American Community Survey. Results showed that compared with those treated in hospitals with neither PHSP nor telehealth postdischarge services, beneficiaries treated in hospitals with PHSP encountered significantly lower Medicare payment and inpatient and readmission rates. Black patients experienced significantly lower cost, inpatient visits, and readmission rates when treated in hospitals with PHSP and telehealth postdischarge services (coefficient = −0.051, *P* < 0.001; incidence rate ratio [IRR] = 0.982, *P* = 0.007; IRR = 0.891, *P* = 0.003). The results of the study demonstrated the importance of combining PHSP and telehealth postdischarge services to improve the efficiency of the health care delivery system and health equity. It is urgent to ensure that PHSs have adequate funding and telehealth infrastructure to support population health.

## Introduction

The COVID-19 pandemic has underscored the urgency to focus on the essential value of public health systems (PHSs) in fostering health equity across the US health care delivery system. PHSs seek to protect and promote the “health of all people in all communities” by “strengthen[ing], support[ing], and mobiliz[ing] communities and partnerships to improve health,” as well as “build[ing] and maintain[ing] a strong organizational infrastructure for public health.”^1^

In addition, the Essential Public Health Services framework's recent revision by the Centers for Disease Control and Prevention (CDC) more centrally features health equity. “Remove systemic and structural barriers that have resulted in health inequities” involves “promoting policies, systems and overall community conditions enab[ling] optimal health for all.”^1^ Numerous studies and government reports, including the CDC, the National Academy of Medicine, the Healthy People initiative, and the White House have advocated strengthening the role of PHSs.^2–5^

PHSs serve as the center of community-engaged health programs and the major source of health care for underserved populations with limited health care access.^6,7^ PHS's activities are more likely to reflect the community health needs^8,9^ and improve patients' trust and engagement in health care.^10,11^ Indeed, evidence has shown that integrating PHSs with the health care system can improve health care quality,^12^ care coordination,^13^ reduce health care cost,^14^ and reduce racial and ethnic disparities in health care^15^ and health.^16,17^

During public emergencies, such as the COVID-19 pandemic, robust partnerships between health care systems and PHSs reflect the community resilience not just through resources of health care and social services but also through the trust and social networks that have been embedded in the communities for decades.

PHS integration and care coordination can be successfully achieved through health information technology (HIT) systems.^18–21^ The 2020 National Health IT Priorities for Research has acknowledged how HITs can improve the care coordination for vulnerable patients with complex health problems and allow providers better access to health information.^22^ The partnership between the Mount Sinai Health System and Contessa (Amedisys, Inc.) shows an example of PHS integration with HIT systems. This integrated system facilitated care coordination and offered home-based care that included home health, hospitalization, rehabilitation, and palliative care at home.^23^

As Chen et al stated, HITs could promote “both horizontal multi-sector integration (across health care providers, home health agencies, nursing homes, long-term care facilities, community partners, public health systems, housing providers, transportation services, etc.) and vertical-level integration (across programs, local networks and health systems, state health systems, and federal policies),”^18^ and contribute to “the Quadruple Aim of improving health care access, quality, reducing cost, and improving health equity.”^24^

The objectives of this article are to examine (1) racial and ethnic disparities in being treated by hospitals with PHS partnership (PHSPs), HIT postdischarge functionalities, and the combination of PHSP and telehealth postdischarge services and (2) the association between PHSP and telehealth postdischarge services and racial and ethnic disparities in Medicare total payment, readmission rates, and inpatient visits. This study focused on hospital-based PHSP and telehealth postdischarge functions among the aging population.

Hospital settings provide critical health care resources for the aging and racial and ethnic minority underserved populations. Hospitals can reduce racial and ethnic disparities by providing timely health care, identifying high-need and high-cost patients, and ensuring the continuation of health care in primary care settings postdischarge.^25,26^ Given PHS's promise to reduce health disparities as well as improve care coordination and HIT infrastructures, the hypothesis is that PHSP coupled with HITs postdischarge has the potential to improve the efficiency of the health care system through care coordination and system integration and reduce racial and ethnic disparities.

## Methods

### Data

The main data sets were 2017 Centers for Medicare and Medicaid Services Medicare (CMS) 100% inpatient claims data and Medicare Beneficiary Summary File (MBSF). These data were linked by beneficiary IDs. Using the Medicare provider IDs, the data were also merged with the American Hospital Association (AHA) Annual Survey to obtain hospital characteristics, hospital-based telehealth measures, and PHSP status. Finally, using beneficiaries' zip codes, this study linked the American Community Survey (ACS) to capture zip-code levels of social determinants of health that were commonly used in the literature.

This study focused on community-dwelling Medicare fee-for-service (FFS) beneficiaries aged 65 years and older, and who had at least 1 hospital visit in 2017. Older adults with Medicare Advantage or Medicare and Medicaid dual eligibility were not included. Non-Hispanic White (White), Black, and Hispanic patients were included.

### Outcome measures

Three outcomes were examined. The first 2 outcomes were created based on the measures from the Medicare Beneficiary Summary Cost and Use file: (1) the “total Medicare payments per person per year” is the summation of Medicare payments on major services, including acute inpatient, skilled nursing facility, hospice, home health, hospital outpatient, Part B physician, and Part D Medicare payments, (2) the readmission rate is the total number of 30-day hospital readmissions in the acute inpatient setting per beneficiary in 2017, and (3) the number of inpatient visits is the total number of inpatient visits per beneficiary in 2017.

### Key independent variables

Hospital PHSP was measured using a dichotomous measure defined as 1 if the hospital reported a partnership with local or state public health organizations (ie, health departments, institutes, etc.) or local or state human/social service organizations (ie, food, housing/rental, energy, transportation assistance), and zero as otherwise.

Hospital-based HIT measures were obtained in the Facilities and Services section of the AHA Annual Survey. A dichotomous measure of telehealth postdischarge functions was defined as 1 if the hospital adopted telehealth remote patient monitoring postdischarge services or telehealth remote patient monitoring for ongoing chronic care management, and 0 as otherwise.

This study focused on whether hospitals had developed a partnership with the PHSs and whether they supported telehealth postdischarge. Hence, PHSPs and telehealth postdischarge services were cross-tabulated into 4 categories: hospitals with PHSP and telehealth postdischarge, no PHSP but telehealth postdischarge, PHSP but no telehealth postdischarge, and no PHSP or telehealth postdischarge.

### Other independent variables

Other independent variables at the beneficiary level included race and ethnicity, age, gender, and health indicators. The Research Triangle Institute (RTI) Race Codes were used for the following groupings: non-Hispanic White, Black, and Hispanic patients. Health indicators included heart diseases, diabetes, hyperlipidemia, hypertension, and asthma obtained from the MBSF. Covariates at the hospital level included teaching status, type of controls (for-profit, nonfor-profit, and government), and bed size.

Area level variables included rurality index using the Core-Based Statistical Areas, the US Office of Management and Budget. Also, a dichotomous measure of whether the hospital offered the telehealth service was defined as 1 if the hospital adopted telehealth consultation and office visits, eICU, stroke care, or psychiatric and addiction treatment, and 0 otherwise. A dichotomous measure for partnerships was defined as 1 if hospitals had partnerships with other stakeholders, such as nonprofit organizations, faith-based organizations, health insurance companies, or other organizations, and 0 otherwise.

Consistent with the literature, the following measures of social determinants of health were included: high school education, poverty as the percentage of people in poverty level for the zip code, and the proportion of Black people in that zip code using the ACS 5-year (2011–2015) average data. Results are presented controlling for these measures of social determinants of health that had been widely used in the literature. Alternatively, models were also tested that controlled for the area deprivation index. Results were similar and available upon request.

### Analysis

The unit of analysis was at the beneficiary–hospital level. Approximately 80% of beneficiaries visited the same hospital, 16% were admitted in 2 hospitals, 3% were admitted in 3 hospitals, and 1% were admitted in more than 3 hospitals. Beneficiary–hospital level data were created to account for situations when beneficiaries visited multiple hospitals and adjusted for the number of records in the regression analysis.

Likelihoods of being treated in hospitals that have developed partnerships with PHSs and have implemented telehealth postdischarge, and beneficiary-, hospital-, and geographic-characteristics were presented and compared by beneficiaries' races and ethnicities. Multivariate regressions then were applied to estimate the associations between hospital PHSP + telehealth postdischarge and outcomes of interests. The study controlled for the beneficiary-, hospital-, and community-level characteristics already presented and applied state-fixed effect analysis in the regressions. Interaction terms were then used between race and ethnicity (Blacks and Hispanics) and PHSP + telehealth postdischarge in the regressions to test different associations by race and ethnicity.

The study employed the generalized linear model with log link and gamma variance distribution to estimate the total Medicare payments and the negative binomial regressions to estimate the rates of readmissions and inpatient visits. The analysis tested different model specifications (eg, with or without community characteristics or state fixed effects; with or without county-level measures of health care resources obtained from the Area Health Resources File) and regressions (eg, linear probability models and logistic regressions for having any readmission, linear regressions of the log of payment) as sensitivity analyses. Results were similar and are available upon request. This study was approved by the Institutional Review Board of the University of Maryland at College Park.

## Results

**FIG. 1.**
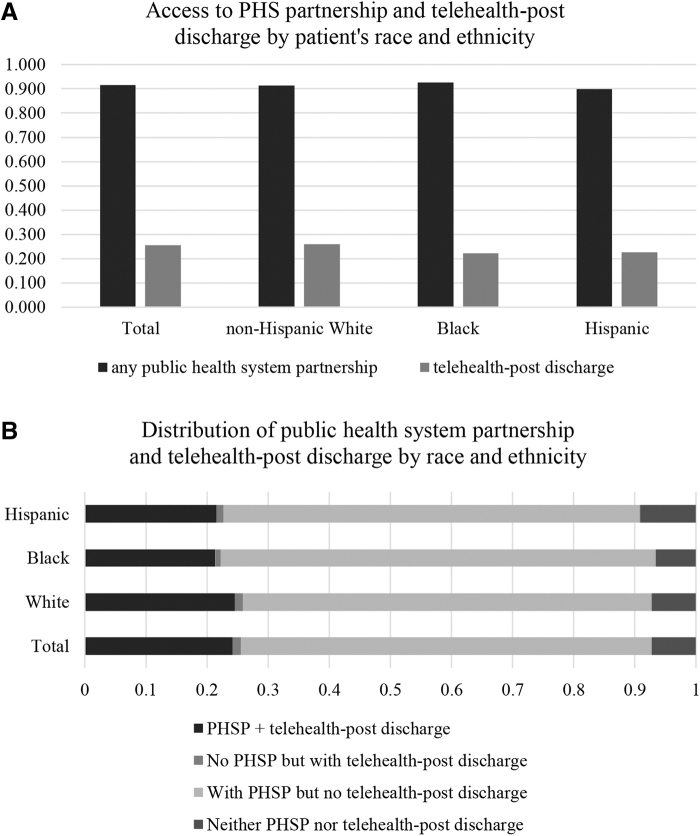
Distribution of PHS partnership **(A)** and telehealth postdischarge services **(A & B)** by race and ethnicity. PHSP, Public health system partnership.

Compared with White beneficiaries, Black and Hispanic Medicare FFS beneficiaries were less likely to be treated in hospitals that provided telehealth postdischarge services (Fig. 1A). Higher percentages of Black beneficiaries were treated in hospitals that had PHSPs but no telehealth postdischarges (Fig. 1B). A higher percentage of Hispanic beneficiaries were treated in hospitals with neither PHSP nor telehealth postdischarge services (9.1% of Hispanics, vs. 6.6% of Black and 7.2% of White patients).

Table 1 presented summary statistics of beneficiary-, hospital-, and community-level characteristics. Black and Hispanic beneficiaries, compared with White beneficiaries, had higher total Medicare payments, more inpatient visits per beneficiary, and readmissions. They were more likely to be aged 65–74 years. Black beneficiaries had significantly higher rates of having heart diseases, diabetes, hypertension, and asthma. Hispanic beneficiaries had higher rates of having diabetes, hypertension, and asthma. Compared with White patients, Black and Hispanic patients were more likely to live in areas with high poverty rates and low education levels.

**Table 1.** Characteristics of Beneficiaries, Hospitals, and Communities by Race and Ethnicity

	Total		Non-Hispanic White		Black			Hispanic		
	*N* = 2,218,233		*N* = 1,990,781		*N* = 151,705			*N* = 75,747		
	Mean	SD	Mean	SD	Mean	SD	*P*	Mean	SD	*P*
Beneficiary characteristics										
Total Medicare payment	31351.050	34376.710	31101.960	33324.810	33739.490	44960.420	<0.001	33113.850	36818.760	<0.001
No. of inpatient visits	1.526	1.039	1.515	1.018	1.641	1.235	<0.001	1.579	1.143	<0.001
No. of readmissions	0.181	0.574	0.177	0.564	0.222	0.667	<0.001	0.202	0.641	<0.001
Age (years)										
Age 65–74	0.490	0.500	0.480	0.500	0.578	0.494	<0.001	0.573	0.495	<0.001
Age 75–84	0.347	0.476	0.352	0.478	0.301	0.459	<0.001	0.304	0.460	<0.001
Age 85 and higher	0.163	0.370	0.168	0.374	0.120	0.326	<0.001	0.123	0.329	<0.001
Female	0.509	0.500	0.508	0.500	0.529	0.499	<0.001	0.490	0.500	<0.001
Chronic conditions										
Heart diseases	0.414	0.493	0.409	0.492	0.485	0.500	<0.001	0.412	0.492	0.0985
Diabetes	0.365	0.481	0.346	0.476	0.532	0.499	<0.001	0.525	0.499	<0.001
Hyperlipidemia	0.732	0.443	0.735	0.442	0.713	0.452	<0.001	0.718	0.450	<0.001
Hypertension	0.864	0.343	0.857	0.350	0.943	0.232	<0.001	0.873	0.333	<0.001
Asthma	0.104	0.306	0.102	0.303	0.127	0.333	<0.001	0.113	0.317	<0.001
Hospital characteristics										
No teaching	0.779	0.415	0.785	0.411	0.687	0.464	<0.001	0.790	0.407	0.0014
Hospital control										
For profit	0.100	0.300	0.095	0.293	0.109	0.312	<0.001	0.208	0.406	<0.001
Not for profit	0.799	0.401	0.805	0.396	0.779	0.415	<0.001	0.689	0.463	<0.001
Government	0.101	0.301	0.100	0.300	0.112	0.315	<0.001	0.104	0.305	0.001
Bed size										
Bed numbers ≤50	0.047	0.211	0.049	0.216	0.020	0.139	<0.001	0.042	0.200	<0.001
Bed numbers 50–200	0.227	0.419	0.233	0.423	0.172	0.377	<0.001	0.193	0.394	<0.001
Bed numbers ≥200	0.726	0.446	0.719	0.450	0.808	0.394	<0.001	0.765	0.424	<0.001
Telehealth treatment	0.620	0.485	0.621	0.485	0.604	0.489	<0.001	0.607	0.488	<0.001
Other partnership	0.957	0.204	0.957	0.204	0.962	0.191	<0.001	0.946	0.226	<0.001
Completely rural area	0.045	0.207	0.046	0.210	0.037	0.189	<0.001	0.032	0.177	<0.001
Adjacent to a metro area	0.177	0.382	0.187	0.390	0.085	0.279	<0.001	0.102	0.303	<0.001
Metro	0.778	0.416	0.767	0.423	0.878	0.327	<0.001	0.865	0.341	<0.001
Community characteristics										
% Population with high school degrees in the community above 50% of the population median	0.501	0.500	0.526	0.499	0.269	0.444	<0.001	0.296	0.457	<0.001
% Population living in poverty in the community above 50% of the population median	0.500	0.500	0.476	0.499	0.735	0.441	<0.001	0.650	0.477	<0.001
% Black population in the community above 50% of the population median	0.499	0.500	0.464	0.499	0.941	0.235	<0.001	0.540	0.498	<0.001

This study focused on community-dwelling Medicare FFS beneficiaries aged 65 years and above, and who had at least 1 hospital visit in 2017. Elderly patients with Medicare Advantage or Medicare and Medicaid dual-eligible patients were not included. After applying the exclusion criteria already mentioned, there was a study population of 3,029,983 beneficiaries. After merging the AHA and obtaining the PHSP and telehealth measures, the final sample size was 2,142,486, including 1,990,781 White, 151,705 Black, and 74,998 Hispanic Medicare FFS beneficiaries. For the readmission regression, the total sample size was 2,126,030, given missing values of readmission; Measures: (1) the “total Medicare payments per person per year” is the summation of Medicare payments on major services, including acute inpatient, other inpatient hospital, skilled nursing facility, hospice, home health, hospital outpatient, ambulatory surgery center, anesthesia, Part B drug, evaluation and management, Part B physician, other procedure, imaging, test, other Part B carrier, and Part D Medicare payments; (2) the readmission rate is the total number of 30-day hospital readmissions in the acute inpatient setting per beneficiary in 2017; and (3) the number of inpatient visits is the total number of inpatient visits per beneficiary in 2017.

AHA, American Hospital Association; PHSP, public health systems partnership; SD, standard deviation.

Table 2 presented state fixed-effect results after controlling for beneficiary-, hospital-, and community-level characteristics. Results of the total Medicare payments show that, compared with those treated in hospitals with neither PHSP nor telehealth postdischarge services, beneficiaries treated in hospitals with no PHSP but with telehealth postdischarge encountered higher total Medicare payment (coefficient = 0.058, *P* < 0.001), whereas beneficiaries treated in hospitals with PHSPs but no telehealth postdischarge services encountered lower Medicare payment (coefficient = −0.07, *P* < 0.001).

**Table 2.** Association of Public Health Systems Partnership + Telehealth Postdischarge and Racial and Ethnic Disparities in Medicare Payment, Inpatient Visits, and Readmission

	Total Medicare payment				Inpatient visit				Readmissions			
	Coefficient	95% CI		*P*	IRR	95% CI		*P*	IRR	95% CI		*P*
White	Reference				Reference				Reference			
Black	0.010	−0.010	0.031	0.318	1.029	1.017	1.041	<0.001	1.106	1.032	1.185	0.004
Hispanic	−0.029	−0.052	−0.006	0.015	1.009	0.996	1.022	0.195	1.007	0.931	1.089	0.870
Neither PHSP nor telehealth postdischarge	Reference				Reference				Reference			
PHSP and telehealth postdischarge	0.003	−0.004	0.010	0.361	0.956	0.952	0.959	<0.001	0.960	0.936	0.984	0.001
No PHSP but with telehealth postdischarge	0.057	0.045	0.069	<0.001	1.009	1.002	1.016	0.014	1.130	1.085	1.178	<0.001
With PHSP but no telehealth postdischarge	−0.070	−0.077	−0.064	<0.001	0.940	0.936	0.943	<0.001	0.904	0.883	0.925	<0.001
PHSP and telehealth postdischarge × Black	−0.051	−0.075	−0.027	<0.001	0.982	0.968	0.995	0.007	0.891	0.825	0.963	0.003
No PHSP but with telehealth postdischarge × Black	−0.058	−0.118	0.001	0.053	0.977	0.944	1.011	0.176	0.849	0.703	1.025	0.088
With PHSP but no telehealth postdischarge × Black	−0.034	−0.055	−0.012	0.002	0.988	0.976	1.000	0.059	0.940	0.875	1.010	0.093
PHSP and telehealth postdischarge × Hispanic	−0.037	−0.065	−0.008	0.011	0.999	0.983	1.015	0.931	1.028	0.937	1.128	0.559
No PHSP but with telehealth postdischarge × Hispanic	−0.055	−0.128	0.018	0.138	1.002	0.963	1.043	0.909	0.990	0.796	1.230	0.925
With PHSP but no telehealth postdischarge × Hispanic	−0.036	−0.061	−0.012	0.004	0.990	0.976	1.004	0.164	1.030	0.947	1.120	0.496

Generalized linear model with log link and gamma variance distribution was used to estimate the total Medicare payments and the negative binomial regressions to estimate the rates of readmissions and inpatient visits. State fixed effects were applied. Covariates presented in Table 1 were controlled for in the regressions.

IRR, incidence rate ratio; PHSP, public health systems partnership.

In the payment regression, the interaction terms of PHSP + telehealth postdischarge and Black (coefficient = −0.051, *P* < 0.001) and Hispanic (coefficient = −0.037, *P* = 0.011) patients were significantly negative. The interaction terms with PHSP but no telehealth postdischarge and Black (coefficient = −0.034, *P* = 0.002) and Hispanic (coefficient = −0.036, *P* = 0.004) patients were also significantly negative.

Results in Table 2 showed that Black patients encountered more inpatient and readmission visits than White patients. Hispanic patients also had higher inpatient and readmission visit rates, but the results were not significant after controlling for all the covariates. Results showed that compared with those treated in hospitals with neither PHSP nor telehealth postdischarge, patients treated in hospitals with a PHSP had a lower incidence rate of inpatient use (incidence rate ratio [IRR] = 0.956, *P* < 0.001) and readmissions (IRR = 0.960, *P* = 0.001).

Patients treated in hospitals with no PHSP but with telehealth postdischarge, however, reported higher inpatient (IRR = 1.009, *P* = 0.014) and readmission rates (IRR = 1.130, *P* < 0.001). The IRR of the interaction term between PHSP and telehealth postdischarge and Black was significantly lower than 1 (IRR = 0.982, *P* = 0.007), indicating a decreased risk of having an inpatient visit. Similar trends were observed in the number of readmissions (IRR = 0.891, *P* = 0.003).

## Discussion

These results suggested that hospitals with PHSPs were associated with lower average total Medicare payments. Black and Hispanic patients treated in hospitals with PHSPs, with or without telehealth postdischarge, encountered lower total Medicare payment. This study showed that combined PHSP and telehealth postdischarge was more effective at reducing inpatient visits and readmissions, especially for Black beneficiaries. More research is needed to understand patterns of inpatient and readmission among Hispanic patients.

The results demonstrate the importance of combining PHSP + telehealth postdischarge to improve efficiency in the health care delivery system and the community infrastructure. The PHSP may be helping to address more social determinants of health indicators such as transportation or food insecurity, and reduce health care costs and readmissions.

There is an argument on whether HITs alone can reduce or increase health disparities. Studies have advocated ensuring that “the future of telemedicine removes disparities in health care access and outcomes instead of exacerbating them.”^27^ Meanwhile, the study noted that “public health practice can shed light on how to address health inequities at the neighborhood level by using a data-driven approach, collaborating with communities, and designing policies with equity in mind.”^26^

A recent study delineated the mechanism through which HITs can be designed to reduce structural racism and discrimination by addressing institutionalized racism, social determinants of health, and system-level barriers in the health care delivery system.^23^ Established evidence has demonstrated that PHSPs can reduce racial and ethnic disparities. Findings of this study suggested that combined PHSs + HITs can be effective to reduce disparities.

Authors speculated that the services provided through PHSs are more likely to address social determinants of health. Many health systems have engaged in initiatives to develop partnerships to address food insecurity through home-delivered meals, transportation, and housing needs.^28^ CalvertHealth Medical Center in southern Maryland implemented a transportation assistance program and observed a 9% reduction in readmission rates.^29^ Working with PHSs, HITs can be designed to reflect community culture and promote patient engagement.^30^

HITs can serve as a useful tool to connect patients with social services or community programs.^31^ PHSP can be successfully achieved by leveraging HIT systems that encompass interoperability to support data sharing with organizations outside of their agencies and potentially can break down long-standing silos between health care providers, community-based organizations, social service agencies, and the public health sector. For example, University of Wisconsin Hospitals and Clinics use the platform ConnectRx to connect eligible patients to community health workers to connect patients with social services.^32^

Through PHSP, HITs can also be designed to improve patient engagement through customized design of patient portals and improve communication by reflecting patient's cultural background and preferences. Meanwhile, PHSs become particularly critical to coordinate care, provide health care access, and promote patient education. The National Academy of Medicine report noted the priorities for health system transformation and PHS's role in remediating disparities and improving health equity.^4^

This study has several limitations. First, the study examined the rather broad combination of PHSP and telehealth postdischarge functions. Future studies can focus on specific mechanisms that can work effectively for a specific population. Second, this analysis focused on the hospital-based PHSPs and telehealth postdischarge services, given the data availability. The model developed in the study, however, can be modified and applied to primary care settings. Third, this study focused on the community-dwelling residents. The role of HITs and care coordination with the help of PHSs in the postacute care settings (eg, skilled nursing facilities, home health agencies) cannot be neglected.

Fourth, the race variables provided by the RTI were used. Detailed measures that separate race and ethnicity can be helpful to understand better the health care utilization patterns among the Hispanic population, and future studies should also include Asian and Native patients. Finally, this study focused the overall expenditures. Future studies can further examine the role of PHSP on access and utilization of primary care and preventive services and other services that target whole-person care and population health.

### Policy implications

Building healthy and sustainable aging-friendly health care structures requires population-based integrated care initiatives at the health care system level. Value-based payment models, such as the Accountable Care Organizations (ACO), and the ACO REACH,^33^ use shared-risk models to urge health systems to address social determinants of health and focus on whole-person care. Such models have the potential to leverage HIT to partner with PHS to promote population health.

More recently (November 1, 2022), the CMS issued the Physician Fee Schedule final ruling, which seeks to promote innovation and care coordination through the Medicare Shared Savings Program, the largest ACO in the United States.^34^ This ruling includes changes to advance overall value-based care on growth, alignment, and equity for ACOs, including HIT infrastructure and telehealth programs, and centers on accountable care between providers and patients. Passage of such rulings highlights increasing recognition for how shared risk models have potential for better patient care.

Emerging evidence shows that PHSPs can improve the efficiency of the health care delivery system and health equity. Measuring community and PHSPs can be used as one of the quality evaluations and reimbursement incentives. There is an urgent need to understand how to integrate PHSs into the health care delivery system and quantify the cost saving and any cost saving associated with reduced health disparities. It is imperative that PHSs have adequate funding and HIT support to be well prepared and positioned to respond to H1N1 (swine flu), Zika, Ebola, COVID-19, and future public emergencies.
